# Carotenoids as natural functional pigments

**DOI:** 10.1007/s11418-019-01364-x

**Published:** 2019-10-01

**Authors:** Takashi Maoka

**Affiliations:** Research Institute for Production Development, 15 Shimogamo, Morimoto Cho, Sakyoku, Kyoto, 606-0805 Japan

**Keywords:** Carotenoids, Natural pigments, Biosyntheses, Metabolism, Function

## Abstract

Carotenoids are tetraterpene pigments that are distributed in photosynthetic bacteria, some species of archaea and fungi, algae, plants, and animals. About 850 naturally occurring carotenoids had been reported up until 2018. Photosynthetic bacteria, fungi, algae, and plants can synthesize carotenoids de novo. Carotenoids are essential pigments in photosynthetic organs along with chlorophylls. Carotenoids also act as photo-protectors, antioxidants, color attractants, and precursors of plant hormones in non-photosynthetic organs of plants. Animals cannot synthesize carotenoids de novo, and so those found in animals are either directly accumulated from food or partly modified through metabolic reactions. So, animal carotenoids show structural diversity. Carotenoids in animals play important roles such precursors of vitamin A, photo-protectors, antioxidants, enhancers of immunity, and contributors to reproduction. In the present review, I describe the structural diversity, function, biosyntheses, and metabolism of natural carotenoids.

## Introduction

### Structure of carotenoids

**Fig. 1:**
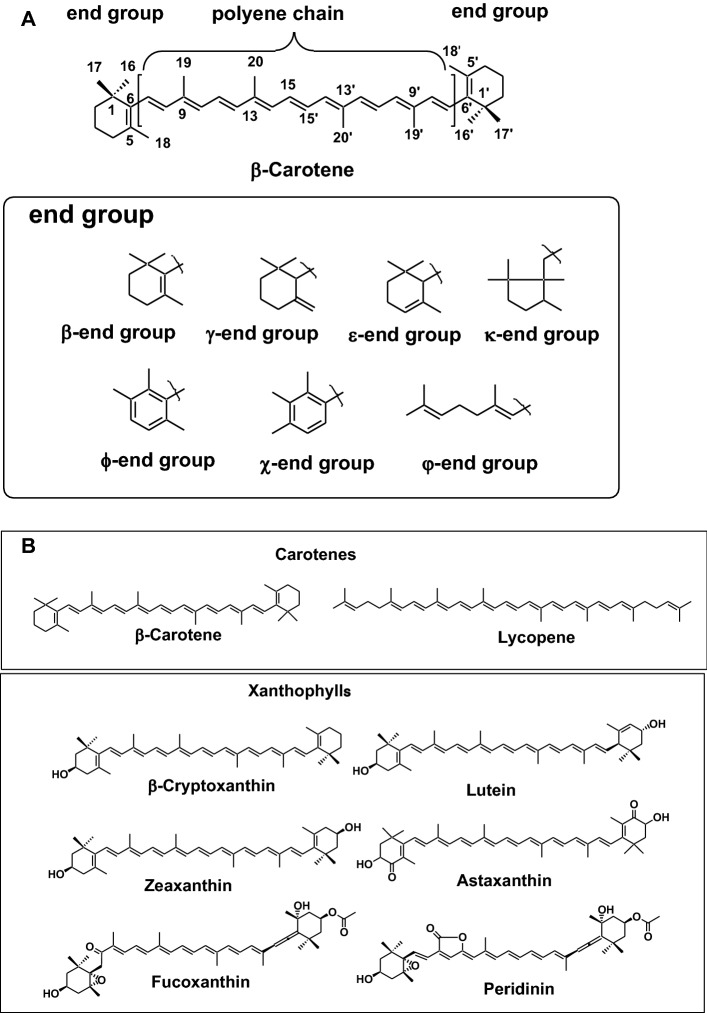
**a** Basic structures of carotenoids and end groups. **b** Structures of typical carotenes and xanthophylls

Carotenoids are tetraterpene pigments, which exhibit yellow, orange, red and purple colors. Carotenoids are the most widely distributed pigments in nature and are present in photosynthetic bacteria, some species of archaea and fungi, algae, plants, and animals. Most carotenoids consist of eight isoprene units with a 40-carbon skeleton. Their general structures commonly consist of a polyene chain with nine conjugated double bonds and an end group at both ends of the polyene chain. The structures of the polyene chain and end groups of carotenoids are shown in Fig. 1a [1]. Carotenoids are divided into two groups: carotenes and xanthophylls. Carotenes, such as *α*-carotene, *β*-carotene, *β*,*ψ*-carotene (*γ*-carotene), and lycopene, are hydrocarbons. About 50 kinds of carotenes are present in nature [1]. On the other hand, xanthophylls, such as *β*-cryptoxanthin, lutein, zeaxanthin, astaxanthin, fucoxanthin, and peridinin, are carotenoids containing oxygen atoms as hydroxy, carbonyl, aldehyde, carboxylic, epoxide, and furanoxide groups in these molecules. Some xanthophylls are present as fatty acid esters, glycosides, sulfates, and protein complexes. Structures of xanthophylls show marked diversity. About 800 kinds of xanthophylls have been reported in nature up until 2018 [1, 2]. Figure 1b shows structures of typical carotenes and xanthophylls. Most carotenoids have 40-carbon skeleton (C40 carotenoid). Some carotenoids have a 45- or 50-carbon skeleton, which are called higher carotenoids. About 40 kinds of higher carotenoids are present in some species of archaea. On the other hand, carotenoids composed of carbon skeletons with fewer than 40 carbons are called apocarotenoids. About 120 kinds of apocarotenoids are present in some species of plants and animals as degradation products of C40 carotenoids [1, 2].

### History of carotenoid research in natural product chemistry

In the early part of the nineteenth century, carotenoids were found in paprika (1817), saffron (1818), annatto (1825), carrots (1831), and autumn leaves (1837). In 1906, Zwet succeeded in the separation of carotene, xanthophyll and chlorophyll from green leaves using column chromatography. In the 1930s, Karrer and Khun elucidated the structures of *β*-carotene and lycopene. Furthermore, they found that *β*-carotene was a precursor of vitamin A. They won the Nobel Prize in chemistry for this work. Subsequently, structures of lutein, zeaxanthin, and astaxanthin were revealed by their groups. These structural studies were based on the oxidative degradation of carotenoids with KMnO_4_, and structures were analyzed using elemental analysis. In the 1950s, the Zechmeister group studied *E*/*Z* (*cis*–*trans*) isomerization of carotenoids. In the 1960s, the Weedon group and Liaaen-Jensen group elucidated the structure of fucoxanthin and peridinin, respectively, using NMR and MS spectrometry [3]. Since the first structural elucidation of* β*-carotene by Kuhn and Karrer in 1928–1930, about 750 naturally occurring carotenoids had been reported up until 2004 [1]. Improvements of analytical instruments such as NMR, MS, and HPLC have made it possible to perform the structural elucidation of very minor carotenoids in nature [2]. Annually, several new structures of carotenoids are being reported. Our research group has performed the structural elucidation and analysis of naturally occurring carotenoids using NMR, MS, MS/MS, and LC/MS [2] over the last decade. About 100 kinds of natural carotenoids were reported from 2004 to 2018 [2]. Synthetic studies of carotenoids revealed the stereochemistry of several complex structures of natural carotenoids such as peridinin, fucoxanthin, crassostreaxanthin B, and cucurbitaxanthin A [4].

## Carotenoids in photosynthetic bacteria, some species of fungi, algae, and plants

### Carotenoid biosynthesis

**Fig. 2:**
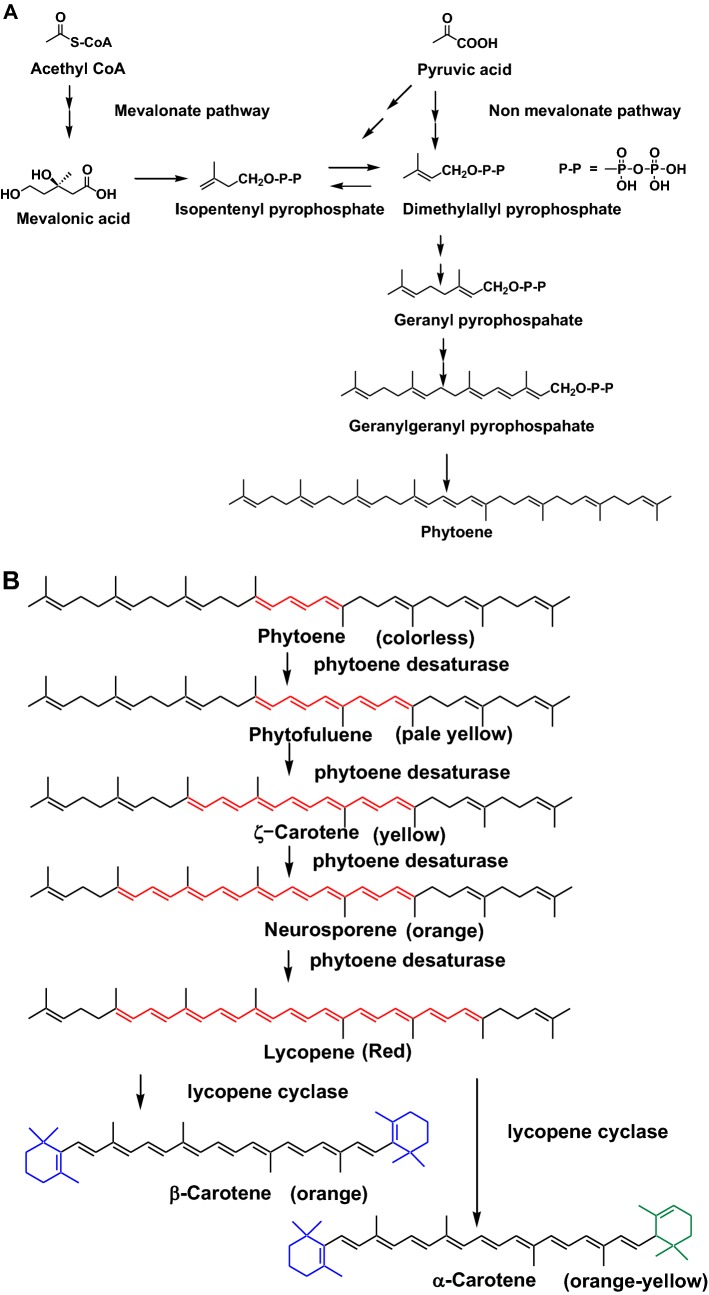
**a** Carotenoid biosynthetic pathways (formation of phytoene). **b** Desaturation of polyene chain and cyclization of end groups

Basic carotenoid biosynthetic pathways are indicated in Fig. 2a and b. The first step, dimethylallyl pyrophosphate is formed from acetyl CoA or pyruvic acid through mevalonate pathway or non-mevanolate pathway, respectively. Then, phytoene, with a C40 carotenoid skeleton, is formed from dimethylallyl pyrophosphate through geranyl pyrophosphate and geranylgeranyl pyrophosphate (Fig. 2a). Phytoene is a colorless carotenoid with three conjugated double bonds. Phytoene is stepwisely desaturated to form lycopene via phytofluene, *ζ*-carotene, and neurosporene by phytoene desaturase. Lycopene cyclases produce carotenoids with cyclic terminal end groups such as *α*-carotene and *β*-carotene, as shown in Fig. 2b. Several xanthophylls are produced by carotene hydroxylases, ketolases, and epoxidase. These carotenoid biosynthetic pathways have been comprehensively revealed by enzymatic and genetic studies [5].

### Carotenoids in photosynthetic organs of plants

Carotenoids are essential compounds along with chlorophylls in photosynthetic bacteria, algae, and plants and are involved in photosynthesis and photo-protection. Carotenoids harvest light energy and transfer this energy to chlorophylls through singlet–singlet excitation transfer. This singlet–singlet transfer is a lower energy state transfer used during photosynthesis. Carotenoids absorb excessive energy from chlorophylls through triplet–triplet transfer and release excessive energy by polyene vibration. The triplet–triplet transfer is a higher energy state essential in photo-protection. Reactive oxygen species such as singlet oxygen, hydroxy radicals, and superoxide anion radicals are produced from oxygen and light during photosynthesis. Carotenoids with more than eleven conjugated double bonds show a marked capacity to quench singlet oxygen. The mechanism for quenching singlet oxygen is a physical reaction. Carotenoids take up thermal energy from singlet oxygen and release this energy by polyene vibration.

**Fig. 3:**
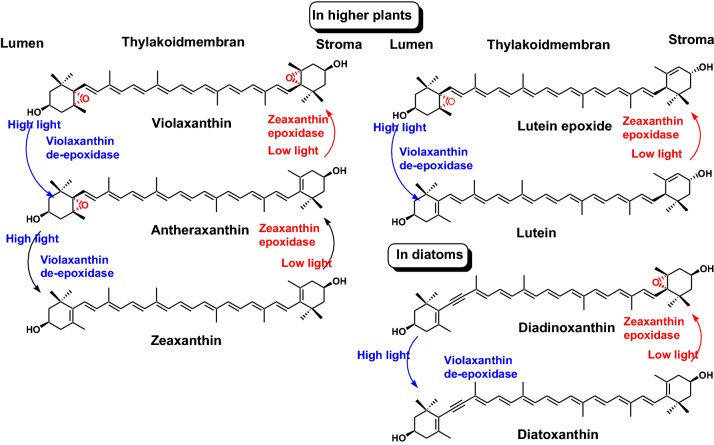
Xanthophyll cycles

The xanthophyll cycle involves the enzymatic removal of epoxy groups from xanthophylls (violaxanthin, antheraxanthin, and lutein epoxide) to create the so-called de-epoxy xanthophylls (zeaxanthin and lutein). These enzymatic cycles were found to play a key role in stimulating energy dissipation within light-harvesting antenna proteins by non-photochemical quenching, a mechanism to reduce the amount of energy that reaches the photosynthetic reaction centers. Non-photochemical quenching is one of the main ways to protect against photoinhibition. In higher plants, xanthophyll cycles consist of violaxanthin–antheraxanthin–zeaxanthin. During light stress, violaxanthin is converted to zeaxanthin via the intermediate antheraxanthin, which plays a direct photo-protective role acting as a lipid-protective antioxidant and by stimulating non-photochemical quenching within light-harvesting proteins. This conversion of violaxanthin to zeaxanthin is medicated by the enzyme violaxanthin de-epoxidase, while the reverse reaction is performed by zeaxanthin epoxidase (Fig. 3) [6]. Lutein epoxide and lutein are members of xanthophylls cycles in higher plants. In diatoms, the xanthophyll cycle consists of diadinoxanthin and diatoxanthin (Fig. 3) [6].

### Carotenoids in non-photosynthetic organs of plants

Carotenoids are also present in non-photosynthetic organs of plants such as fruits, pericarps, seeds, roots, and flowers. Carotenoids in these none-photosynthetic organs show structural diversity and are formed by secondary metabolic reactions, such as oxidation, the cleavage of polyene chains, and (*Z*/*E*) (*cis*–*trans*) isomerization [5, 6]. Carotenoids in non-photosynthetic organs act as photo-protectors, antioxidants, color attractants, and precursors of plant hormones.

Many fruits and seeds turn red or purple during the ripening stage. This color change is due to the formation of carotenoids and/or anthocyanins. For example, the color of the pericarp of tomato turns from greenish-yellow to deep red during the ripening stage. This color change is due to the conversion of phytoene to lycopene in the pericap of the tomato. Phytoene (colorless), which is the major carotenoid in greenish-yellow tomato, is converted to phytofluene (pale yellow), *ζ*-carotene (yellow), neurosporene (orange), and lycopene (red) by phytoene desaturase, as shown in Fig. 2B [5, 6]. Lycopene is a carotenoid with some of the strongest singlet oxygen quenching and photo-protection activities [5, 6].

**Fig. 4:**
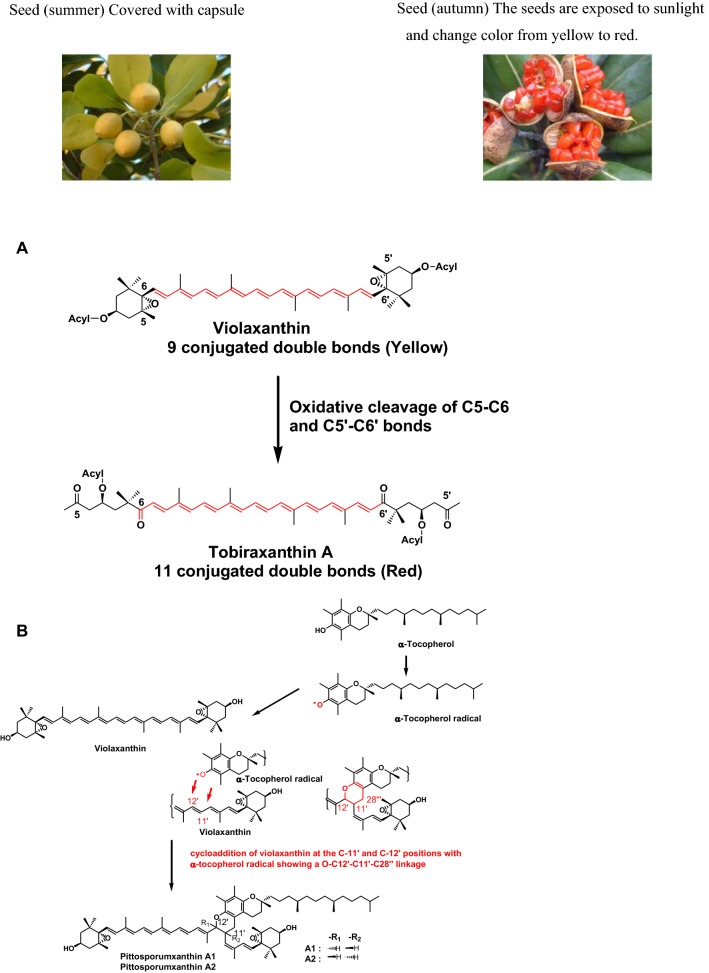
**a** Formation of tobiraxanthin and **b** formation of pittosporumxanthins in the seeds of *Pittosporum tobira*

*Pittosporum tobira* (Tobera in Japanese) is a small, slender, evergreen tree growing in southern Japan. In summer, the seeds have a pale yellow color and are covered with a capsule. In autumn, the seeds are exposed to sunlight and change color from yellow to red. The major carotenoid in the yellow seeds is violaxanthin, with a pale yellow color, and related epoxy carotenoids. On the other hand, the major carotenoid in the red seeds is a series of red seco-carotenoids named as tobiraxanthin A, B, and C. During the autumn season, violaxanthin is converted to tobiraxanthin A by oxidative cleavage of C5–C6 and C5′–C6′ bonds, as shown in Fig. 4a. Tobiraxanthin A shows an approximately 30-nm longer wavelength shift than violaxanthin because of the introduction of two conjugated carbonyl groups at C6 and C6′ in the polyene chain of violaxanthin. Therefore, tobiraxanthin A shows strong activity to quench singlet oxygen induced by sunlight. Furthermore, the red color of the seed acts as an attractant for birds to eat seeds to disperse them. Therefore, red seco-carotenoids act as antioxidants, photo-protectors, and color attractants in *P. tobira* (Fig. 4a) [2, 7].

Novel carotenoid and *α*-tocopherol complexes named pittosporumxanthins were also isolated from the red-colored seeds of *P. tobira* [2, 8, 9] by our research group. Pittosporumxanthin A1 and A2 are diastereomeric pairs of the cycloaddition product of violaxanthin at the C-11′ and C-12′ positions, with *α*-tocopherol (Fig. 4b). The mechanism of carotenoid and *α*-tocopherol complex formation in the seeds of *P. tobira* may be considered as follows: an *α*-tocopherol radical is formed from *α*-tocopherol by the quenching or scavenging of reactive oxygen species in the seed. The *α*-tocopherol radical itself becomes a pro-oxidant and causes oxidative damage. Therefore, carotenoids such as violaxanthin take up *α*-tocopherol radicals through the formation of adduct products. This suggests that carotenoids can quench phenoxy radicals by reacting with these polyene chains [2, 8, 9].

Carotenoids also participate in different types of cell signaling. They are able to signal the production of abscisic acid, which regulate plant growth, seed dormancy, embryo maturation and germination, cell division and elongation, floral growth, and stress responses. Carotenoids are also precursors of some aroma compounds [6].

## Carotenoids in animals

In general, animals do not synthesize carotenoids de novo, and so those found in animals are either directly obtained from food or partly modified through metabolic reactions. The major metabolic conversions of carotenoids found in these animals are oxidation, reduction, translation of double bonds, oxidative cleavage of double bonds, and cleavage of epoxy bonds [5, 6, 10].

It is well-known that carotenoids that contain unsubstituted *β*-ionone rings such as *β*-carotene, α-carotene, *β*-cryptoxanthin. and *β,ψ*-carotene (*γ*-carotene) are precursor of retinoids and are called pro-vitamin A. Furthermore, carotenoids in animals play important roles such as photo-protectors, antioxidants, enhancers of immunity, and contributors to reproduction. Several animals use carotenoids as signals for intra-species (sexual signaling, social status signaling, and parent–offspring signaling) and inter-species (species recognition, warning coloration, mimicry, and crypsis) communication [5, 6, 10].

### Food chain and metabolism of carotenoids in aquatic animals

Aquatic animals contain various carotenoids that show structural diversity. Aquatic animals obtain carotenoids from foods such as algae and other animals and modify them through metabolic reactions. Many of the carotenoids present in aquatic animals are metabolites of* β*-carotene, fucoxanthin, peridinin, diatoxanthin, alloxanthin, and astaxanthin [2, 10, 11]*.*

**Fig. 5:**
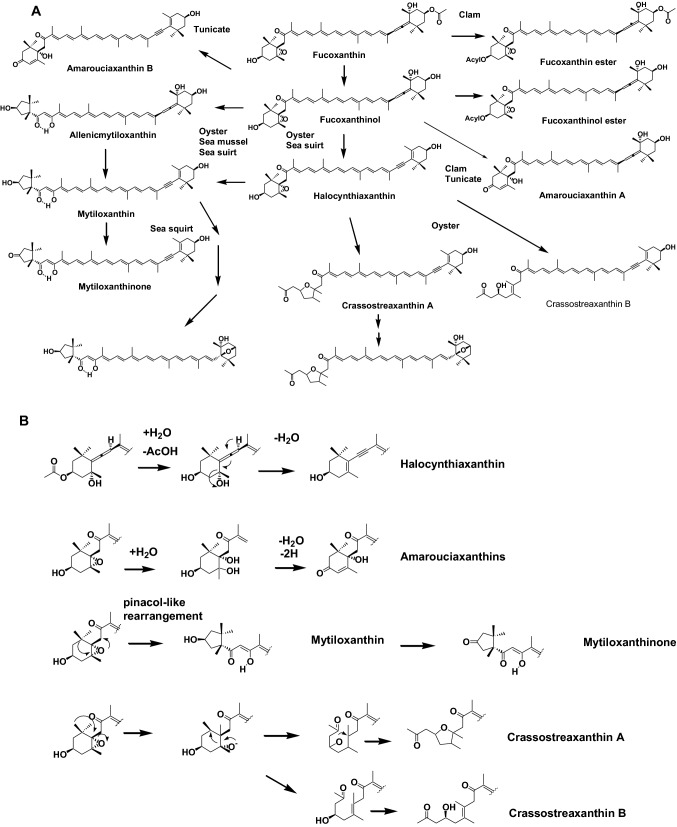
**a** Metabolic pathways of fucoxanthin in bivalves and tunicates. **b** Metabolic conversion mechanisms of end groups of fucoxanthin in bivalves and tunicates

Bivalves (oysters, clams, scallops, mussels, and ark shells) and tunicates (sea squirts) are filter feeders. They feed on micro-algae such as diatoms, dinoflagellates, blue-green algae, and green algae and obtain carotenoids from these dietary sources. The major carotenoid in diatoms is fucoxanthin. Fucoxanthin has several functional groups, such as allenic bond, epoxide, carbonyl, and acetyl groups. Therefore, metabolites of fucoxanthin in bivalves and tunicates show structural diversity, as shown in Fig. 5a. The major metabolic conversions of fucoxanthin found in these animals are the conversion of allenic bond to acetylenic bond, hydrolysis cleavage of epoxy group, and oxidative cleavage of epoxy group, as shown in Fig. 5b [2, 10].

**Fig. 6:**
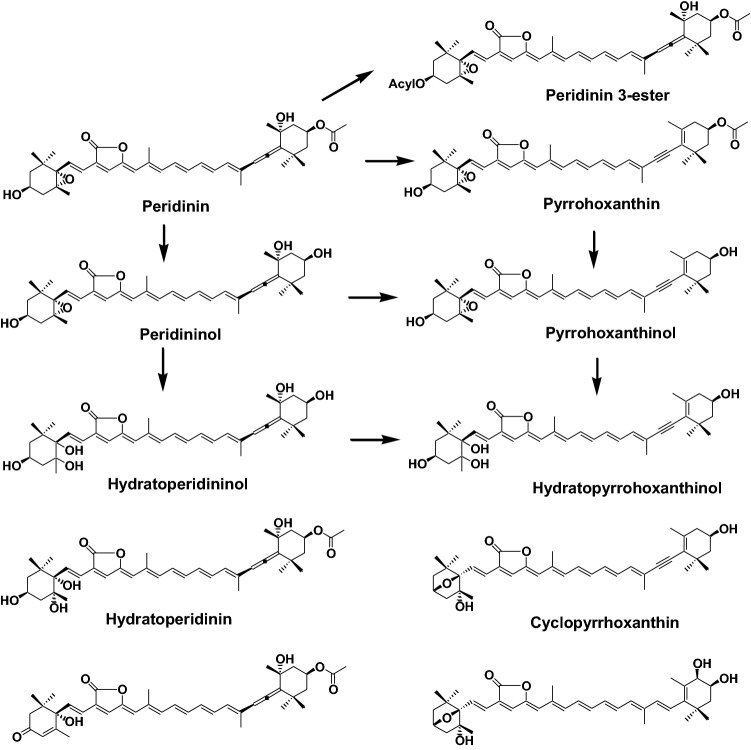
Metabolic pathways of peridinin in bivalves and tunicates

Peridinin, with its C37 carbon skeleton, is a major red carotenoid in dinoflagellates. Peridinin also has several functional groups, such as an allenic bond, epoxide, and a lactone ring. As well as fucoxanthin, peridinin is also converted to several metabolites in bivalves and tunicates, as shown in Fig. 6 [2, 10].

**Fig. 7:**
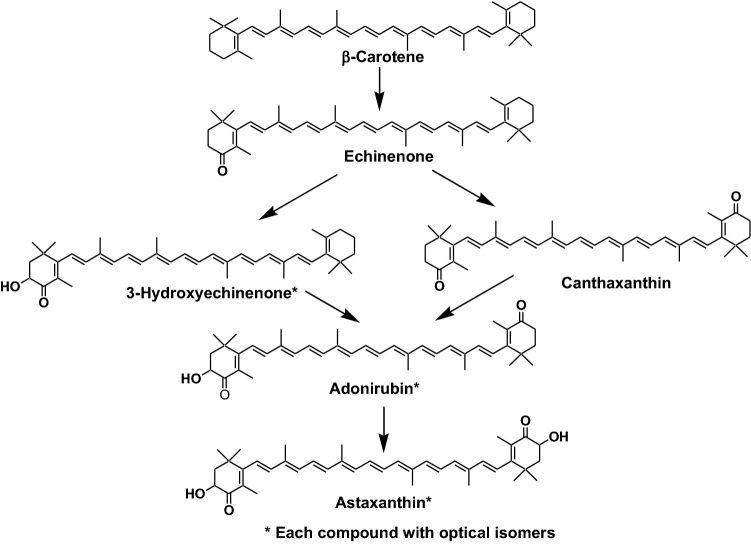
Oxidative metabolism of* β*-carotene in crustaceans

Astaxanthin is a characteristic marine carotenoid in crustaceans (shrimps and crabs). Many crustaceans can synthesize astaxanthin from* β*-carotene, ingested in dietary algae, via echinenone, 3-hydroxyechinenone, canthaxanthin, and adonirubin, as shown in Fig. 7 [5, 11]. In many crustaceans, hydroxylation at C-3 (C-3′) in the 4-oxo-*β*-end group is non-stereo-selective. Therefore, astaxanthin and related carotenoids with a 3-hydroxy-4-oxo-*β*-end group, present in crustaceans, are comprised of a mixture of these optical isomers [5, 11].

**Fig. 8:**
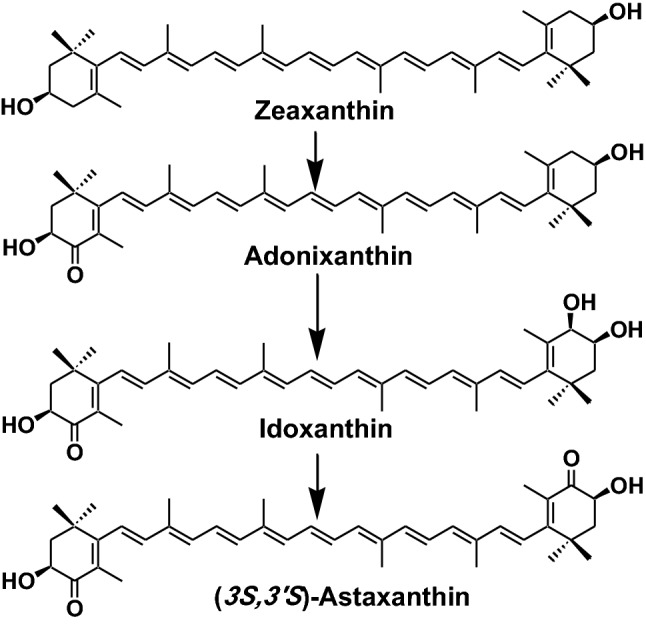
Metabolic conversion of zeaxanthin to (3*S*,3′*S*)-astaxanthin in Cyprinidae fish

Carp, crucian carp, and goldfish belonging to Cyprinidae can convert zeaxanthin to (3*S*,3′*S*)-astaxanthin via adonixanthin and idoxanthin (Fig. 8). Therefore, spirulina, which contains zeaxanthin as the major carotenoid, is used for pigmentation in red carp and goldfish [10, 11].

**Fig. 9:**
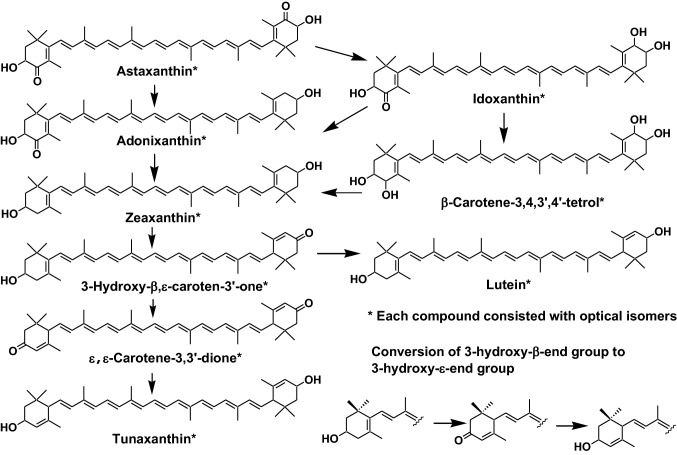
Reductive metabolic pathway of astaxanthin in marine fish

On the other hand, several marine fish (red sea bream, cod, tuna, and yellow-tail) and Salmonidae fish (salmon and trout) cannot synthesize astaxanthin from other carotenoids such as *β*-carotene and zeaxanthin [5, 10, 11]. Therefore, astaxanthin present in these fish originates from dietary zooplankton belonging to Crustacea such as krill. So, astaxanthin is used for pigmentation in red sea bream and salmon. The bright yellow color of the fin and skin of several marine fish is due to the presence of tunaxanthin (*ε,ε*-carotene-3,3′-diol). Tunaxanthin is metabolized from astaxanthin via zeaxanthin, as shown in Fig. 9 [5, 10, 11]. Carotenoids with a 3-oxo-ε-end group such as 3-hydroxy-*β*,ε-caroten-3′-one and ε,ε-carotene-3,3′-dione are key intermediates in this metabolic conversion.

### Biological function of carotenoids in marine animals

As described above, marine animals convert dietary carotenoids and accumulate them in these organs. Through these metabolic conversions, antioxidative and photo-protective activities of carotenoids are increased.

For example, many marine invertebrates such as crustaceans convert *β*-carotene to astaxanthin and accumulate it in integuments, carapaces, eggs, and ovaries. Through the metabolic conversion, the carotenoid changes its color from yellow (*β*-carotene) to red (astaxanthin). Astaxanthin in marine invertebrates sometimes forms a carotenoid protein complex and is a red, blue, or purple color. These colors may serve to camouflage the animals in the prevailing undersea light conditions, serve as general photoreceptors, or provide protection against possible harmful effects of light. Furthermore, through this metabolic conversion, the antioxidant effects of carotenoids such as the quenching of singlet oxygen, inhibiting lipid peroxidation, and protection against photo-oxidation are enhanced. Therefore, astaxanthin in these animals acts as an antioxidant and prevents oxidative stress [6].

**Fig. 10:**
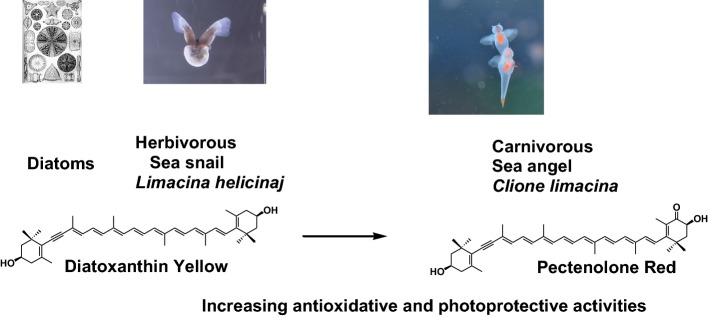
Food chain and metabolism of carotenoids in sea angel

The next example involves carotenoids in the gonads of the sea angel *Clione limacine* [12]. The sea angel is a small, floating sea slug belonging to the class Gastropoda. It inhabits under the drift-ice in the sea of Okhotsk and is exposed to strong sunlight. Its body is gelatinous and transparent. On the other hand, its gonads and viscera are a bright orange-red color due to the presence of carotenoids. The sea angel is carnivorous and feeds exclusively on a small sea snail *Limacina helicina*, which is herbivorous and feeds on micro-algae such as diatoms and dinoflagellates. Therefore, carotenoids produced by micro-algae are made available to the sea angel through *L. helicina* in the food chain. *L. helicina* directly absorbs carotenoids such as diatoxanthin from dietary algae and accumulates them without metabolic modification. On the other hand, the sea angel oxidatively metabolizes ingested diatoxanthin from *L. helicina* to pectenolone, as shown in Fig. 10 [12]. By introducing a carbonyl group at C-4′ in diatoxanthin, the carotenoid changes color from yellow to red and shows enhanced antioxidative and photo-protective activities. Therefore, the sea angel accumulates pectenolone in the gonads as an anti-oxidant and a photo-protector [12].

The red carotenoid mytiloxanthin is a metabolite of fucoxanthin present in shellfish and tunicates. Dietal fucoxanthin from diatoms is metabolized to mytiloxanthin via fucoxanthinol and halocynthiaxanthin in shellfish and tunicates, as shown in Fig. 5a. Through this metabolic conversion, antioxidative activities such as singlet oxygen quenching, scavenging of hydroxy radicals, and inhibition of lipid peroxidation of carotenoids are increased. Mytiloxanthin showed almost the same excellent antioxidative activity as that of astaxanthin [13]. Therefore, it was concluded that marine animals metabolize dietary carotenoids to a more active antioxidative form and accumulate them in their bodies and reproductive organs.

**Fig. 11:**
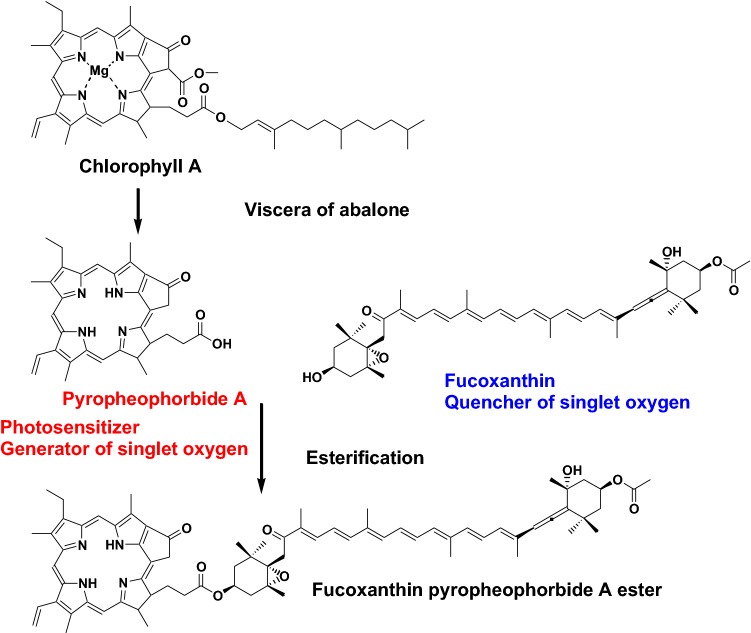
Novel carotenoid pyropheophorbide A ester from abalone

A novel fucoxanthin pyropheophorbide A ester (Fig. 11) was isolated from the viscera of the abalone *Haliotis diversicolor aquatilis*. The major food sources of abalone are macro-algae such as brown algae, which contain fucoxanthin as the major carotenoid. Pyropheophorbide A is a metabolite of chlorophyll A in the viscera of abalone. This carotenoid pyropheophorbide A ester might be formed from fucoxanthin and pyropheophorbide A by esterase in the abalone viscera. It is well-known that pyropheophorbide A is a photosensitizer that generates singlet oxygen from ground-state molecular oxygen in the presence of light. On the other hand, carotenoids are excellent quenchers of singlet oxygen and they prevent photo-oxidation. Therefore, it is interesting that compounds acting as singlet oxygen generators and quenchers are linked with esterified bonds. Indeed, fucoxanthin pyropheophorbide A ester shows weaker singlet oxygen generation than pyropheophorbide A [14].

### Carotenoids in terrestrial animals

As with aquatic animals, most terrestrial animals cannot synthesize carotenoids de novo, and so must obtain them from their diet. Therefore, carotenoids in terrestrial animals mainly originate from plants that they feed on. Many of the carotenoids present in terrestrial animals are *β*-carotene, *β*-cryptoxanthin, lutein, zeaxanthin, and their metabolites [5, 6].

#### Carotenoids in insects and spiders

**Fig. 12:**
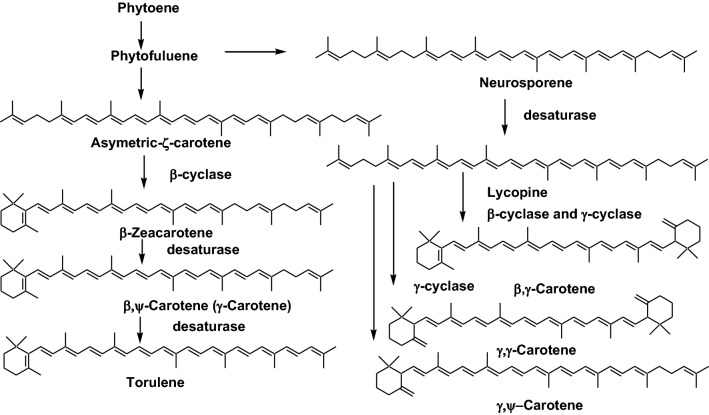
Carotenoid biosynthetic pathways by horizontal transfer genes from fungi and symbiotic bacteria in aphid and whitefly

Insects are the most diverse group of animals. Therefore, carotenoids in insects show structural diversity. Many of the carotenoids present in insect are *β*-carotene, *β*-cryptoxanthin, lutein, and zeaxanthin, which originate from their food, and their metabolites. On the other hand, the aphid and whitefly can synthesize carotenoids de novo by carotenoid biosynthesis genes that are acquired via horizontal gene transfer from fungi or endosymbiotic bacteria. These insect synthesize *β*-zeacarotene, *β,ψ*-carotene (*γ*-carotene), torulene, *β,γ*-carotene, and *γ,γ*-carotene by carotenoid biosynthesis genes transferred from endosymbiotic bacteria (Fig. 12) [15]. Furthermore, blue-green aphids synthesize polycyclic quinines using genes of the endosymbiotic bacterium *Rickettsiella* [16]. Therefore, aphids make their own carotenoids and quinines by horizontal transfer genes from fungi or symbiotic bacteria for coloration, depending on the environmental context. These aphid carotenoids are also accumulated in beetles and dragonflies through the food chain.

Stick insects change their body color from green to red for camouflage in autumn. In summer, stick insects accumulate chlorophyll and *β*-carotene from dietary green leaves and show a green body color. In autumn, they convert *β*-carotene to 3,4,3′,4′-tetradehydro-*β,β*-carotene-2,2′-dione, with 15 conjugated double bonds system, which exhibits deep red color. This carotenoid is also accumulated in eggs for reproduction [17]. A series of ketocarotenoids, 3-hydroxyechinenone, adonirubin, and astaxanthin, was identified in the two-spotted spider mite *Tetranychus urticae*. In response to long nights and lower temperatures, female spider mites enter a facultative diapause characterized by the cessation of reproduction and a marked change in body color from faint yellow to bright red–orange. This body color change results from the accumulation of ketocarotenoids, like astaxanthin, which has been suggested to protect against the physical stresses of overwintering.

A recent investigation revealed that carotenoid cyclase/synthase and carotenoid desaturase genes, which may be responsible for the conversion of phytoene to* β*-carotene, were present in the two-spotted spider mite *T. urticae* [18, 19]. Phylogenetic analyses suggest that these carotenoid biosynthetic genes were transferred from fungi into the spider mite genome.

#### Carotenoids in birds

Most of the bright red, orange, and yellow pigments of plumage (feathers) are due to the presence of carotenoids. In birds, carotenoids are an important signal of a good nutritional condition and they are used in ornamental displays as a sign of fitness and to increase sexual attractiveness. Carotenoid-based colors of feathers (plumage) catch the attention of the opposite sex to promote mating. For example, manipulation of the dietary carotenoid supply invokes parallel changes in cell-mediated immune function and sexual attractiveness in male zebra finches [6, 20]. At least ten kinds of carotenoids have been documented in red feathers. Most of these are produced through metabolic modification of dietary precursor compounds. A series of yellow carotenoids with the 3-hydoxy- and/or 3-oxo-ε-end group were also reported in colored feathers of the goldfinch *Carduelis*. They are also metabolized from lutein and zeaxanthin [21].

Recently, Mundy et al. identified genes required for the bright-red coloration that birds use for communication, such as attracting mates. They revealed a genetic link between red coloration and color vision in the zebra finch, and proposed that redness may be an honest signal of mate quality by indicating a bird’s ability to detoxify harmful substances [22]. Carotenoids are also present in frogs, snails, and lizards. These yellow and red colors are due to the presence of carotenoids such as* β*-carotene,* β*-cryptoxanthin, lutein, and astaxanthin.

#### Carotenoids in mammals

It has been reported that mammals are categorized into three groups in terms of their ability to absorb carotenoids. White-fat animals such as pigs, sheep, goats, cats, and rodents do not absorb carotenoids at all or in very small amounts. Yellow-fat animals, such as ruminant cattle and horses exclusively accumulate carotenes and not xanthophylls. The third group, humans and monkeys, accumulate both carotenes and xanthophylls equally well [6].

A feeding experiment revealed that monkeys effectively absorbed not only* β*-carotene but also *β*-cryptoxanthin, lutein, and zeaxanthin into plasma. In the liver, both *β*-carotene and xanthophylls were well-deposited. In the lung, heart, muscle, fat, skin, and brain, less polar carotenoids such as *β*-carotene and *β*-cryptoxanthin, were well-deposited rather than polar xanthophylls such as lutein and zeaxanthin. Namely, the plasma carotenoid profile in monkeys reflected the dietary carotenoid composition, as in humans. Monkeys effectively accumulated not only *β*-carotene but also *β*-cryptoxanthin, lutein, and zeaxanthin in plasma. Interestingly, monkeys were similar with regard to the preferential accumulation of* β*-cryptoxanthin in the blood and brain [23].

**Fig. 13:**
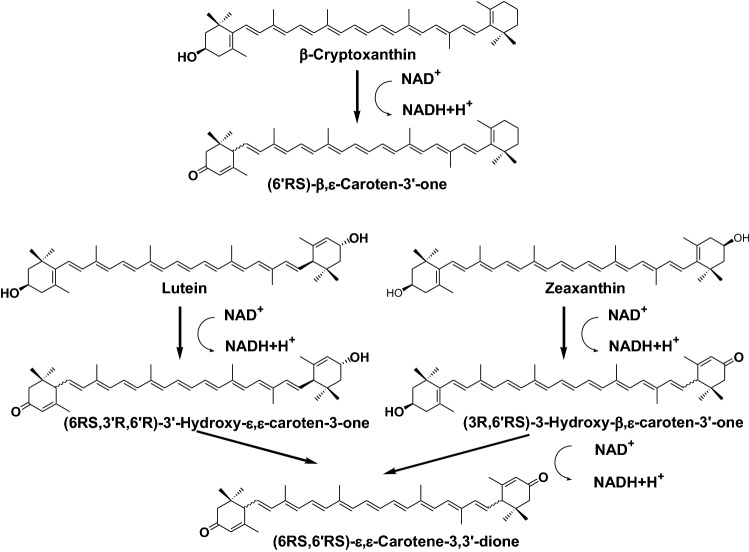
Oxidation pathway of xanthophylls with 3-hydroxy-*β*-end in mammals

Xanthophylls with the 3-oxo-ε-end group, such as *β,ε*-caroten-3′-one, 3-hydroxy-*β,ε*-caroten-3′-one, 3′-hydroxy-ε,ε-caroten-3′-one, and ε,ε-carotene-3,3′-dione, are present in several mammals. Recently, Nagao et al. revealed that carotenoids with a 3-hydroxy-*β*-end group in xanthophylls were oxidized to carotenoids with a 3-oxo-ε-end group via an unstable intermediate with a 3-oxo-*β*-end group by a NAD^+^-dependent dehydrogenase of the mouse liver, as shown in Fig. 13 [24].

#### Carotenoids in humans

About 50 kinds of carotenoids are found in common human foods, and among them, about 20 types ingested from food are found in the blood (plasma or serum). Of these, *β*-carotene, *α*-carotene, lycopene, *β*-cryptoxanthin, lutein, and zeaxanthin have been found to be the major components and make up more than 90% of the total carotenoids [25, 26]. Carotenoids also accumulate in human erythrocytes [23]. Oxidative metabolites of lycopene, lutein, and zeaxanthin are also found in human plasma [23, 25, 26]. Capsanthin, a major carotenoid in paprika, is also absorbed in humans and part of it is metabolized to capsanthone [23]. However, epoxy carotenoids, such as antheraxanthin, violaxanthin, neoxanthin, and lutein epoxide, which are present in vegetables, are not found in human blood. These epoxy carotenoids might be degraded by to the acidic conditions in the stomach [27–29].

Carotenoids ingested from the diet are absorbed by the small intestine. Xanthophyll esters are hydrolyzed by lipase or esterase and absorbed. A part of provitamin A carotenoids are converted into retinal in the mucous of the small intestine by *β*-carotene-15,15′-dioxygenase. Absorbed carotenoids are incorporated into chylomicrons and then transported to the liver and various organs through the blood. All three major lipoproteins: very low-density lipoprotein (VLDL), low-density lipoprotein (LDL), and high-density lipoprotein (HDL), are involved in the transport of carotenoids [28]. Carotenoids can be found in several human organs, such as the liver, adrenal gland, ovaries, skin, lung, testes, prostate, and blood serum. The distribution of carotenoids in human organs shows specificity. Lutein and zeaxanthin are found in the surface of the skin and subcutaneous tissue in an esterified form and act as UV absorbers and quenchers of singlet oxygen [30]. Xanthophylls such as *β*-cryptoxanthin, lutein, and zeaxanthin are found in the brain [31]. In the eye, lutein (*meso*)-zeaxanthin, and zeaxanthin are present as macular pigments [6]. Lycopene accumulates in the prostate [28, 29].

Several investigations have revealed that dietary carotenoids are associated with reduced risks of some cancers and other serious conditions, stimulation of the immune systems and benefits for skin health of humans [6, 28, 29].

In 1981, Peto et al. reported that dietary *β*-carotene reduced human cancer rates [32]. Since then, several epidemiological studies have demonstrated that intake of green-yellow vegetables and fruits, which contain various carotenoids, is associated with a reduced risk of cancer [6, 28, 29]. For example,* β*-cryptoxanthin, which is rich in Satuma mandarin (*Citrus unshiu*), could be associated with a reduced risk of cancer. Intake of lycopene also reduced the risk of prostate cancer [6, 28, 29]. Furthermore, clinical trials have also revealed that the administration of natural multi carotenoids (mixture of α-carotene,* β*-carotene, lutein, and lycopene) and α-tocopherol resulted in significant suppression of hepatoma development in a hepatitis virus-induced cirrhosis patient [33, 34]. Carotenoids were also reported to help prevent cardiovascular disease, diabetes, obesity, and several lifestyle-related diseases and to enhance immunity. Furthermore, carotenoids improve endurance and skin health [6, 28, 29].

## Chemical mechanism of scavenging of active oxygen species by carotenoids

It was reported that the mechanism whereby carotenoids scavenge singlet oxygen was a physical reaction. Namely, carotenoids take up thermal energy from singlet oxygen and release this energy by polyene vibration [6]. However, recent investigations including our research group studies revealed that carotenoids could scavenge reactive oxygen species through chemical reactions.

**Fig. 14:**
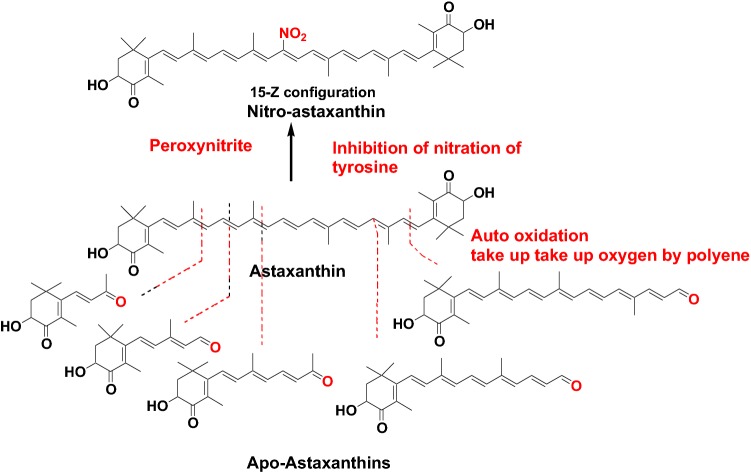
Auto oxidation products of astaxanthin and reaction products of astaxanthin with peroxynitrite

A series of apo-astaxanthin was obtained as out-oxidation products of astaxanthin. These compounds were considered to take up oxygen by polyene in astaxanthin (Fig. 14) [35]. Astaxanthin also forms nito-astaxanthins by the reaction of peroxynitrite and inhibits the nitration of tyrosine in an in vitro model (Fig. 14). These results indicate that astaxanthin is able to capture peroxynitrite and nitrogen dioxide radicals from their molecules to form nitro-astaxanthin and protects against the nitration of tyrosine. Similar results were obtained in cases of* β*-carotene, lutein, zeaxanthin, capsanthin, and fucoxanthin. These results suggest that carotenoids might have higher reactivity with peroxynitrite and/or nitrogen dioxide radicals than phenoic compounds such as tyrosine. Therefore, carotenoids could inhibit the nitration of tyrosine [36, 37].

**Fig. 15:**
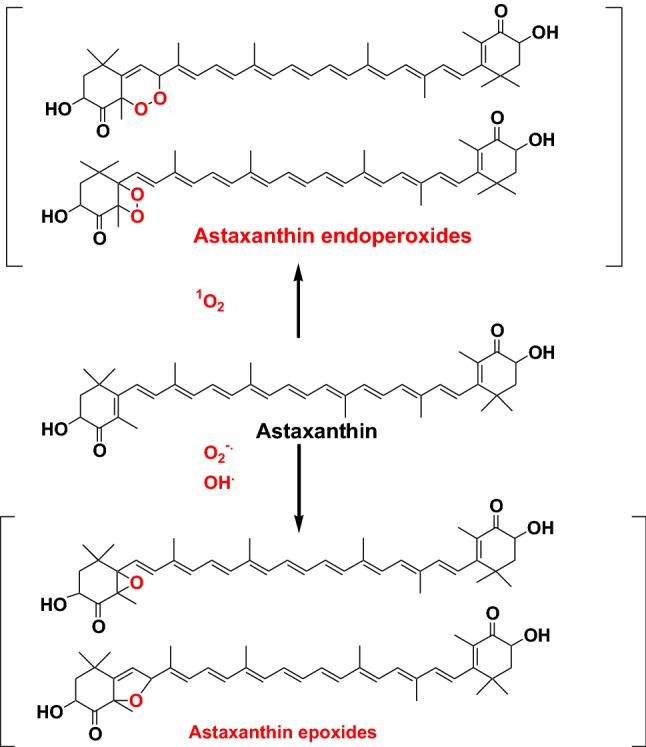
Reaction products of ataxanthin with hydroxy radicals, superoxide anion radicals, and singlet oxygen

Recently we investigated the reaction products of astaxanthin with hydroxy radicals, superoxide anion radicals, and singlet oxygen by LC/PDA ESI–MS and ESR spectrometry. The ESR study revealed that astaxanthin could quench not only singlet oxygen but also superoxide anion radicals and hydroxy radicals. The LC/PDA ESI–MS study revealed that astaxanthin epoxides were major reaction products of astaxanthin with superoxide anion radicals and hydroxyl radicals. Astaxanthin endoperoxides were identified as major reaction products of astaxanthin with singlet oxygen (Fig. 15) [38]. Similar results were also obtained in the case of* β*-carotene, zeaxanthin, and capsanthin [39, 40]. These results suggest that carotenoids could take up singlet oxygen, superoxide anion radicals and hydroxyl radicals by the formation of endoperoxide or epoxide.

## Conclusion

Until 1980, interest in carotenoid research was exclusively focused in the field of natural product chemistry and fisheries science in Japan. Many interesting structural carotenoids were identified in aquatic animals. Since then, several biological functions of natural carotenoids have been established through investigation of natural product chemistry, aquaculture, human-health science, and photosynthesis. Japanese researchers have been contributing to the progress of carotenoids science, technology, and commercial application. Now, carotenoids are well-known to be important for human health, playing roles in the prevention of cancer and lifestyle-related diseases and contributing to the beauty industry. Carotenoids, such as astaxanthin, are therefore industrially produced and used for supplements and cosmetics. The 19th International Symposium on Carotenoids will held in Toyama from July 12 to 17, 2020. I hope for the success of this symposium and further progress in carotenoid science and business in Japan.
